# Foreign Language Teachers’ Emotion Recognition in College Oral English Classroom Teaching

**DOI:** 10.3389/fpsyg.2021.782379

**Published:** 2021-11-03

**Authors:** Yanyun Dai

**Affiliations:** School of Western Languages, Anhui International Studies University, Hefei, China

**Keywords:** emotion recognition, college oral English, foreign language teacher, teaching, emotion recognition in college oral English

## Abstract

One of the significant courses in Chinese universities is English. This course is usually taught by a foreign language instructor. There will, however, necessarily be some communication hurdles between “foreign language teachers” and “native students.” This research presents an emotion recognition method for foreign language teachers in order to eliminate communication barriers between teachers and students and improve student learning efficiency. We discovered four factors of emotion recognition through literature analysis: smile, eye contact, gesture, and tone. We believe that differences in foreign language teachers’ performance in these four areas will have an impact on students’ emotion recognition and, as a result, on their learning efficiency. The influence of the foreign language teacher’s eye contact and gestures is larger (the weight of a single variable accounted for 30% or more) in the decision whether can improve the students’ classroom learning efficiency, according to 43 of the questionnaire data analysis. The second is the tone and smile (the weight of a single variable accounted for between 10 and 20%). Our research contributes to the body of knowledge on emotion recognition in university foreign language teachers by presenting a practical method for recognizing emotion in foreign language teachers. We recommend that college foreign language teachers pay attention to eye and gesture communication with students in English classrooms based on the findings. By enriching the style of emotional expression in class, college language teachers, particularly foreign language teachers, can improve communication, and connection with students.

## Introduction

In China, the majority of universities offer English language courses. Moreover, these courses are all taught by foreign native English speakers. There are many advantages to employing foreign language teachers as lecturers of college English courses. For example, as a native speaker, their pronunciation is very pure, the language logic is very authentic, and the description of transactions, and scenes can be very appropriate (Chen and Lee, 2011). However, it is inevitable for Chinese students to encounter some obstacles in their English learning because the teacher is a foreigner. For example, students with weak English foundation may find it difficult to keep up with the speed of the teacher’s speech and understand the main points of the teacher’s lecture. In China, where Mandarin is the official language, English is not used much as a second language.

In fact, it is an effective way to increase the understanding of foreign language teachers by identifying their emotions (Lorette and Dewaele, 2015). The teacher’s smile, body movements, intonation, etc., can all become the reference way for students to judge context and understand semantics (Gong and Rina, 2021). Emotion recognition refers to that the computer analyses and processes the signals collected from the sensor, so as to get the emotional state of the other party (person) (Cowie et al., 2001). At present, there are two methods for emotion recognition, one is to detect physiological signals such as respiration, heart rhythm, and body temperature, and the other is to detect emotional behaviors such as facial feature expression recognition, voice emotion recognition, and posture recognition (Kwon et al., 2003). In college English class, students can recognize the emotion by observing the facial expression, pronunciation, and posture of foreign language teachers, so as to help them understand the course content.

Many scholars have studied the emotion recognition of foreign language teachers in English classrooms. For example, (Chen and Lee, 2011) proposed an embedded human emotion recognition system to help teachers reduce individual learners’ language learning anxiety. Lorette and Dewaele (2019) studied the relationship between visual, sound, speech, and emotion recognition ability, and multilingual ability in foreign language teaching. Barcelos and Ruohotie-Lyhty (2018) explore the relationship between emotion and belief and how this understanding can be used to support the development of language teachers. The above examples are all studies of different regions. However, in mainland China, emotion recognition is particularly important for foreign language teachers in English classes due to the widespread Chinese learning. And there’s not much research in this area. Therefore, the research question for this study is: What factors are employed to identify emotion recognition in foreign English teachers? What is the relative importance of these variables? In view of this, this paper mainly does the following work. First, we propose the factors that can be used for emotion recognition of foreign language teachers. Secondly, we analyze the influence degree of each factor by R software, nnet package and garson algorithm. Our research has enriched the literature on the field of emotion recognition for foreign language teachers and, in practice, has helped to guide students to assist classroom learning by identifying teachers’ emotions.

The rest of the paper is arranged as follows: the second section describes the work related to emotion recognition of foreign language teachers; the third section presents the analytical method and experimental results; the results are discussed in the fourth section; in the fifth and last section, we put forward the research conclusions, limitations, and possible future research directions.

## Related Work

### Emotion Recognition and Its Role

Professor Rosalind W. Picard from the Media Lab of Massachusetts Institute of Technology first proposed to create a computer system that can sense, recognize, and understand human emotions and make intelligent, sensitive and friendly responses by recognizing emotional signals of human body (Picard, 1997). At present, many studies on emotion recognition focus on text [e.g., (Yoon et al., 2018; Batbaatar et al., 2019)], facial expressions [e.g., (Liliana, 2019; Wang et al., 2020)], and speech recognition [e.g., (Alu et al., 2017; Tao et al., 2018)]. The emergence of these researches, especially the development and application of emotion recognition intelligent system, makes machines have “emotion.” We also make use of scholars’ research results to carry out emotional recognition on foreign language teachers’ teaching posture, facial expressions and other aspects.

Emotion recognition is widely used in human life (Cai et al., 2021). For example, the application of emotion recognition in vehicle driving (Wu et al., 2021) can monitor the state of drivers and identify emotions from their voice, body language and facial expressions, so as to help reduce the occurrence of traffic accidents (Vögel et al., 2018). The application of emotion recognition in the field of elderly care can increase verbal communication and interaction with the elderly and relieve the loneliness of the elderly group (Wang et al., 2018). In addition, (Estrada et al., 2020) introduced emotion recognition into students’ language teaching classes to identify students’ expression skills. It can be seen that the application of emotion recognition in all walks of life has a broad basis. This provides a literature basis for this paper to evaluate the performance of foreign language teachers by using emotion recognition.

### Emotion Recognition Factors for College Foreign Language Teachers

Based on different perspectives and environments, scholars have conducted diversified studies on the influencing factors of emotion recognition of college foreign language teachers. For example, (Unsworth and Mills, 2020) proposed that facial expressions and gestures could be used as evaluation factors for emotion recognition. Similarly, (Tonguç and Ozkara, 2020), (Rieffe and Wiefferink, 2017) also emphasize the important role of facial expressions in emotion recognition. In addition to the above factors, (Haq and Jackson, 2011) proposed that audio and vision should also be used as important reference aspects of emotion recognition.

According to the above literature analysis, emotion recognition is widely used in human life, work, and study. At present, in college language courses, emotion recognition technology has become an important way to assist teachers (Liang et al., 2021) to understand students’ performance [e.g., (Unsworth and Mills, 2020)]. However, previous studies have paid little attention to how students identify emotions through different aspects of foreign language teachers. The research on this issue is very necessary, because it helps students to better understand the teaching content of foreign language teachers.

## Methods and Results

**FIGURE 1:**
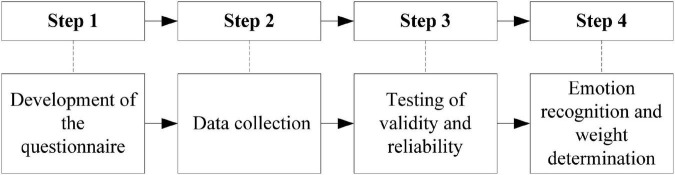
Research steps.

The research process in this paper mainly consists of four steps, as shown in Figure 1. The first step is questionnaire development, the second step is data collection, the third step is the reliability and validity test of the scale and questionnaire, and the fourth step is emotion identification and weight calculation of independent variables.

### Questionnaire Development

Based on the above analysis of the emotion recognition factors of college foreign language teachers, we determined four factors as independent variables of this paper: smile, eye contact, gesture, and tone. We hypothesize that students can effectively identify the emotion of foreign language teachers through the judgment of these four factors, so as to improve their classroom learning efficiency. Therefore, we take students’ classroom learning efficiency as the dependent variable. Accordingly, we developed the scale shown in Table 1. It should be noted that the four independent variables are numerical variables, and students can rate the teacher’s performance in smile, eye contact, gesture, and tone. The score ranges from 1 to 7, and the higher the score, the better the performance of foreign language teachers in this aspect, and vice versa. Whether it is helpful to improve the classroom learning efficiency is classified value, where 1 represents helpful to improve the classroom learning efficiency, 0 represents no help to improve the classroom learning efficiency.

**TABLE 1:** Measurement scale.

**No.**	**Variables**	**Abbreviations**	**Mark**
1	Smile	SM	1–7
2	Eye contact	EC	1–7
3	Gesture	GE	1–7
4	Tone	TO	1–7
5	The improvement of classroom learning efficiency	ICLE	1, 0

### Data Collection

After the questionnaire was designed, we determined the respondents of this study. The students in this survey need to meet the following two requirements: (1) have studied college English courses in the past 1 year; (2) their teacher of this course is a foreigner (native English speaker). Our survey was carried out by filling in offline. A total of 43 students were surveyed during a week (from April 23, 2021 to April 29, 2021). Of the 43 students, 52% are freshmen and 48% are sophomores. The male to female ratio is 4:6. The age is mainly between 19 and 22 years old. 25 students thought that their foreign language teachers performed better in these four variables, which improved their classroom learning efficiency, while 18 students held the opposite view.

### Reliability and Validity Tests

We conducted reliability and validity tests on the scale designed in this paper and the collected questionnaires (Brink, 1991; Roberts and Priest, 2006), and the results are shown in Tables 2, 3. It can be seen from Table 2 that Cronbach’s Alpha coefficient is greater than 0.8, indicating that the scale and questionnaire data have high reliability. The data in Table 3 show that both Kaiser-Meyer-Olkin (KMO) and Bartlett’s test meet relevant thresholds, indicating that our scale and questionnaire data have good validity (Brink, 1991).

**TABLE 2:** Reliability statistics.

**Cronbach’s alpha**	**Cronbach’s alpha based on standardized items**	**N of items**
0.893	0.930	5
		

**TABLE 3:** Kaiser-Meyer-Olkin (KMO) and Bartlett’s test.

**Kaiser-Meyer-Olkin measure of sampling adequacy.**		**0.846**
Bartlett’s test of sphericity	Approx. Chi-Square	177.400
	df	10
	Sig.	0.000

### Emotion Recognition and Weight Calculation

In this paper, we use R software and nnet package to carry out the emotion recognition of foreign language teachers and the weight measurement of the variables. R is a language and operating environment used for statistical analysis and mapping (Ihaka and Gentleman, 1996), as well as a free and open source software belonging to GNU system (Maronna et al., 2019).

Nnet packets are feedforward neural networks and multinomial log-linear models that can be used to process forward and back propagation data until convergence (Ripley et al., 2016). In this paper, we set the parameters of the model to: size = 5, rang = 0.1, decay = 5e−2, and maxit = 5,000.

**FIGURE 2:**
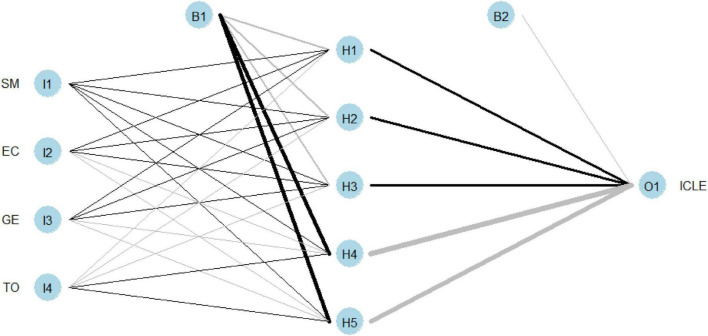
Calculation model.

By drawing the model in R language, we get the calculation model shown in Figure 2. Figure 2 shows that there are five nodes in a single hidden layer.

**FIGURE 3:**
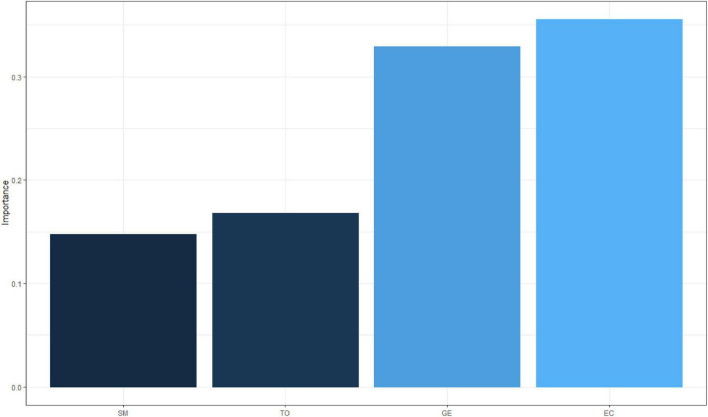
Weight of independent variables.

Then, we used the NeuralNetTools package (Beck, 2018) to obtain the relative importance (weight) of input variables (smile, eye contact, gesture, and tone) in the neural network (Zhao et al., 2020; Chu et al., 2021; You et al., 2021; Zhang et al., 2021) through garson algorithm (Ghanizadeh et al., 2020). As shown in Figure 3, it can be found that the foreign language teacher’s eye contact and gesture have a greater influence on the decision of whether to improve students’ classroom learning efficiency (the weight of each variable is above 30%), followed by tone and smile (the weight of each variable is between 10 and 20%).

## Discussion

In this paper, we analyze the factors that can be used for emotion recognition through literature review. We then identify the specific factors of emotion recognition by university foreign language teachers, which are smile, eye contact, gesture, and tone of voice, respectively. Furthermore, we use the nnet package of R and garson algorithm to draw the emotion recognition model of college foreign language teachers and determine the weight of each variable. Our study found that eye contact and gestures have a large impact on English teaching by college foreign language teachers (each weighing more than 30% for a single variable), while tone and smile have a relatively small impact (each weighing between 10 and 20% for a single variable). Based on the above findings, we propose the following implications.

### Theoretical Implications

From the perspective of literature, research content, and methods, our research has three theoretical implications. Firstly, this paper enrichis the literature on emotion recognition of foreign language teachers in the university. Mandarin is the official language in China. English, as a widely used language in the world, is the second language of most people in China. Hence, in many Chinese universities, more and more foreign language teachers come from english-speaking countries. In addition to the positive effects, it will inevitably bring students a certain degree of language learning disabilities. This paper aims to improve students’ learning efficiency from four aspects: smile, eye contact, gesture, and tone. In addition, the majority of the previous studies are based on the perspective of teachers to identify students’ emotions (e.g., Estrada et al., 2020; Unsworth and Mills, 2020; Liang et al., 2021). However, in line with (Lorette and Dewaele, 2015) and (Gong and Rina, 2021), we believe that, from the perspective of students, identifying the lecturer’s emotion can assist students better understand the subject delivered in English class by the teacher. This paper tries to identify the emotion of foreign language teachers from the perspective of students. Therefore, this paper enriches the literature on emotion recognition for foreign language teachers.

Second, we propose specific factors that can be used to identify foreign teachers’ emotion. Emotion recognition is uncertain and may be disturbed by different factors in different environments. Based on the context of college English courses, this paper takes foreign language teachers as research subjects and identifies four factors (smile, eyes contact, gesture, and tone) that can be used to identify their emotions. This conclusion has certain theoretical reference value for the peer research. But there is no denying that our conclusions are suggestive and instructive. We look forward to exploring different emotion recognition factors in different courses, different environments, and different objects.

Thirdly, this paper introduces nnet package and garson algorithm into the research of emotion recognition of university foreign language teachers, and provides a feasible method of emotion recognition. This method is easy to operate in R software and is applicable to both small sample and large sample data. It is worth mentioning that we can clearly calculate the weights of different independent variables. Therefore, this paper extends the application of nnet package and agrson algorithm in emotion recognition of college foreign language teachers.

### Practical Implications

Our findings are likely to benefit teachers and students in college English classes. On the one hand, foreign English teachers can adjust their body movements or language habits in class to improve teaching efficiency based on our research findings; on the other hand, students can assist in understanding course content based on foreign English teachers’ performance in the four factors examined in this paper. Specifically, there are two practical implications of this paper. First of all, college foreign language teachers should pay attention to eye contact and gesture communication with students in English classes. Our study found that eye contact and gestures of foreign language teachers have a significant influence on the decision of whether to improve students’ classroom learning efficiency (each variable accounts for more than 30% of the weight). This suggests that rich and varied eye and gesture communication can help students deepen their understanding of the knowledge in English class and improve their learning efficiency. The majority of foreign language teachers come from native English-speaking countries, so students’ expectations on tone are not very strong. The reason may be that the vast majority of students think that having good pronunciation and oral English is the basic skills that foreign language teachers should have. The eye contact and gestures, as the auxiliary language expressions of foreign language teachers, have a great influence on students.

Second, in college language teaching, especially foreign language teachers can enhance communication and interaction with classmates by enriching the way of emotional expression in class. This is conducive to the improvement of students’ learning efficiency. In this paper, the four factors of emotion recognition, smile, eye contact, gesture, and tone pair, all have an impact on the improvement of students’ learning efficiency. Therefore, college language teachers need to recognize the importance of emotional communication in the classroom and assist teaching through smiles, eye contact, gestures, and tones of voice.

## Conclusion

One of the essential courses in Chinese colleges is English, which is usually taught by a foreign language teacher. However, contact between “foreign language teachers” and “native Chinese students” will necessarily be hampered. This research presents an emotion recognition method for foreign language teachers in order to eliminate communication barriers between teachers and students and improve student learning efficiency. We discovered four factors of emotion recognition through literature analysis: smile, eye contact, gesture, and tone. We believe that disparities in foreign language teachers’ performance in these four aspects will have an impact on students’ emotion recognition and, as a result, on their learning efficiency. The influence of the foreign language teacher’s eye contact and gestures is larger (the weight of a single variable accounted for 30% or more) in the decision whether can improve the students’ classroom learning efficiency, according to 43 of the questionnaire data analysis. The second is the tone and smile (the weight of a single variable accounted for between 10 and 20%). Our research contributes to the body of knowledge on emotion recognition in university foreign language teachers by presenting a practical method for recognizing emotion in foreign language teachers.

We recommend that college foreign language teachers pay attention to eye and gesture communication with students in English classrooms based on the findings. By enriching the style of emotional expression in class, college language teachers, particularly foreign language teachers, can improve communication, and connection with students.

To be clear, our research has a lot of flaws. For example, because our study’s sample size is rather small, we can expand it in the future. Second, whether there are disparities in foreign language teachers’ emotional performance in the classroom among universities and localities, and how students react to such differences. These are intriguing questions that should be investigated further. Third, this article simply evaluates the foreign language teacher’s smile, eye contact, gestures, and tone. Dress and teaching posture (standing or sitting) are also worth investigating further. Fourth, many English teachers at Chinese universities are actually Chinese. Do their issues with emotion recognition differ from those of foreign language instructors? This is something that needs to be looked at more. Finally, there are numerous approaches for recognizing emotions. We can test multiple approaches for identifying foreign language teachers’ emotions and analyze the benefits and drawbacks of each method as well as the disparities in conclusions.

## Data Availability Statement

The datasets presented in this study can be found in online repositories. The names of the repository/repositories and accession number(s) can be found below: https://github.com/appdevcode/Emotion_Teaching.

## Ethics Statement

The studies involving human participants were reviewed and approved by the Anhui International Studies University. The patients/participants provided their written informed consent to participate in this study.

## Author Contributions

YD: conceptualization, methodology, software, writing, data curation, and investigation.

## Conflict of Interest

The author declares that the research was conducted in the absence of any commercial or financial relationships that could be construed as a potential conflict of interest.

## Publisher’s Note

All claims expressed in this article are solely those of the authors and do not necessarily represent those of their affiliated organizations, or those of the publisher, the editors and the reviewers. Any product that may be evaluated in this article, or claim that may be made by its manufacturer, is not guaranteed or endorsed by the publisher.
